# Gambling Motives and Offshore Gambling: A Finnish Population Study

**DOI:** 10.1007/s10899-023-10253-8

**Published:** 2023-09-28

**Authors:** Heli Hagfors, Atte Oksanen, Anne H. Salonen

**Affiliations:** 1 Faculty of Social Sciences, Tampere University, Tampere, 33014 Finland; 2 Health and Wellbeing Promotion Unit, Finnish Institute for Health and Welfare, Tampere, Finland; 3 Faculty of Health Sciences, University of Eastern Finland, Tampere, Finland

**Keywords:** Offshore gambling, Domestic gambling, Population study, Problem gambling, Monopoly, Gambling motives, Online gambling

## Abstract

The rise of online gambling has drawn attention towards offshore gambling. Currently there is lack of evidence on reasons and motivations to gamble on offshore gambling sites. This study investigated the general gambling motives of onshore and offshore gamblers, and the reasons to gamble on offshore gambling sites. The study used binary logistic regression model to analyze the data from Finnish Gambling 2019 population survey including adult past-year online gamblers (n = 1,422). The validated measure for problem gambling severity (PGSI, Problem Gambling Severity Index) was used. Furthermore, data-driven qualitative analysis was used to form categories for the reasons to gamble on offshore gambling sites. Offshore gambling was more common among men and younger age groups than among women or older age groups. Offshore gamblers gambled less often for money or worthy causes than onshore gamblers. Furthermore, offshore gamblers had more different types of motives to gamble, they gambled more frequently and had higher problem gambling severity scores (PGSI) than onshore gamblers. Finally, the most common reasons to gamble offshore were: (1) larger game supply and game features, (2) benefits, bonuses, and the usability of the website, and (3) inner motivation. Offshore gambling is characterized with intensity and diversity of gambling behavior and motives, and it poses a risk especially for young men.

## Introduction

Many countries regulate gambling services to prevent gambling harms, crime, and other negative social outcomes (Banks, 2017; Sulkunen et al., 2019). There are great differences between countries how the regulation is arranged from licensing systems to monopoly suppliers. Rapid rise on the popularity of online gambling has drawn the attention to offshore gambling operators which accept online gamblers in contravention to local regulations even though they do not have local licenses (Gainsbury et al., 2018; Hing et al., 2021; Lind et al., 2021). The popularity of offshore gambling raises some concerns as the offshore operators may offer access to products that do not meet local restrictions for gambling or do not follow consumer protection requirements (Forsström & Cisneros Örnberg, 2019; Hing et al., 2021; Podesta & Thomas, 2017). For example, unregulated gambling operators have been associated with difficulties or inability of withdrawing winnings, and frauds, such as identity thefts (Banks, 2012; Griffiths, 2010; Gainsbury et al., 2018; Hing et al., 2021). Moreover, offshore gambling may predispose to exacerbation of gambling problems as offshore sites are accessible 24 h a day with large game variety (Silvennoinen & Vuorento, 2022). Having multiple player accounts makes it more difficult to follow how much has been spent to gambling overall. For those who have voluntarily self-excluded from gambling through licensed operators, offshore gambling sites may pose a risk to breach one’s own self-exclusion (Håkansson & Åkesson, 2022). Offshore gambling has also been linked to trading with high-risk cryptocurrencies (Oksanen et al., 2022).

The reasons for gambling, called gambling motives, are key concept in understanding gambling behavior and potential gambling harm, such as problem gambling. To our knowledge, only a few studies have investigated the reasons to gamble on offshore gambling sites, and no prior study has compared the general gambling motives between online gamblers who use only onshore gambling sites as opposed to gamblers who also use offshore gambling sites. This study aimed to fill this gap with an analysis of offshore and onshore gamblers in Finland.

### Offshore and Onshore Gambling

In this paper, we use the term offshore gambling for those operators which do not hold a valid operating license in a particular jurisdictional area but provide gambling services for online gamblers from that area (Gainsbury et al., 2019; Hing et al., 2021; Podesta & Thomas, 2017). Gambling operators which hold a license for a given jurisdictional area are referred as onshore gambling operators (Podesta & Thomas, 2017). Onshore gambling operators must follow the governmental regulations for marketing and promotion, offer adequate consumer protection tools, and pay taxes for the given jurisdictional area (Gainsbury et al., 2019; Marionneau & Järvinen-Tassopoulos, 2017). Offshore gambling operators compete with onshore operators in the same markets with similar products and services, or with the kind of products and services that regulated sites are unable to offer, but do not pay taxes or follow the local restrictions (Gainsbury et al., 2019). By competing with onshore gambling sites, offshore gambling operators undermine the value of licenses while having an unfair competing advantage. For these reasons, many governments try to channel gambling for their licensed gambling operators.

For some gamblers, offshore gambling sites might seem tempting as they may provide greater variance of different products, payouts, and gambling experiences. Moreover, the restrictions the onshore sites must use might be frustrating for some compared to offshore sites (Costes et al., 2016; Gainsbury et al., 2018). It is also noteworthy, however, that gamblers may not always be aware of the unlicensed status of the gambling site (Hing et al., 2021), as the gambling websites can mimic or copy the design of a licensed gambling website (Griffiths,^2010^). Based on our knowledge, only a few studies have examined the characteristics of those who use offshore gambling sites and their reasons for site selection. Previous studies have associated offshore gambling with younger age (Costes et al., 2016; Gainsbury et al., 2018, 2019) but the associations with gender are somewhat mixed. One study found that offshore gamblers were more often men (Gainsbury et al., 2018) while two studies found offshore gambling to be more common among women (Costes et al., 2016; Gainsbury et al., 2019).

The proportion of problem gambling is higher among offshore than onshore gamblers (Hing et al., 2021), and offshore gambling is also associated with more intense gambling patterns (Costes et al., 2016) and greater variance of game types (Gainsbury et al., 2019). The most common reasons for offshore gambling are greater variance in gambling options, higher payout rates and better prices (Gainsbury et al., 2018; Hing et al., 2021). The ability to pay in local currency and the usability of the website were also listed as reasons for choosing one site over another (Gainsbury et al., 2019). Furthermore, some gamblers might be encouraged to use offshore gambling sites due to betting restrictions that onshore gambling operators place to their gambling accounts if they gamble too successfully (Podesta & Thomas, 2017).

### Motivation, Addiction, and Gambling Motives

The role of motivation has been recognized as an important factor in gambling behavior and addictions (Baumeister, 2016; Binde, 2013; Köpetz et al., 2013). Motivation can be defined as any sort of desire to do something (Baumeister & Vohs, 2007). Baumeister and Vohs (2007) distinguish two forms of motivations; drives and impulses. Drive is a more long-term desire or a recurrent pattern of behavior that heads toward some goal that may not be present at the current situation. Impulse, or a state, is a situational and contextual desire to perform a particular act in a particular occasion. Drive can be seen as trait of a person which is independent of the situations whereas impulse is a product of the interaction between person and a situation. Some situational cue can activate an impulse.

Self-regulation is a concept closely connected to motivation and addiction, which includes setting a goal, finding appropriate means to achieve the goal, rejecting distractions, and managing conflicts (Köpetz et al., 2013). Goals have motivational properties as they represent a desirable end-state which can be achieved through certain action. At the beginning of an addiction, means (such as gambling) are just different pathways to the desirable end-state (e.g., enhancement or relaxation). As time passes, the means are cognitively and emotionally associated with the goal through a process called emotional transfer, and the mean becomes the goal in itself that the individual compulsively chases.

Previous research has associated difficulties in impulse control with addictive behaviors (Ioannidis et al., 2019). Impulsivity is a multifaceted concept which consist of impulsive choice, impulsive action and impulsive personality traits (negative and positive urgency, lack of persistence, lack of planning and sensation seeking) (MacKillop et al., 2016; Whiteside et al., 2005). The tendency to act rash without thinking, difficulty to tolerate negative emotions, and the preference of small immediate rewards rather than large but delayed rewards (delay discounting) are typical characters of impulsive behavior, and may maintain the addiction despite the negative consequences (Amlung et al., 2017; MacKillop et al., 2016).

In order to understand gambling behavior more in depth, researchers have identified psychological reasons for gambling, in other words gambling motives (e.g. Binde, 2013; Stewart & Zack, 2008). Although the possibility to win money is the fundamental motive to gamble, many other reasons to gamble have been recognized (Binde, 2013). Previous studies have identified five motives in addition to monetary motive: enhancement, socializing, challenge, escape and supporting worthy causes (Binde, 2013; Dechant, 2014; Francis et al., 2015; Wardle et al., 2011). According to different population studies (Francis et al., 2015; Pallesen et al., 2020; Volberg et al., 2015; Wardle et al., 2011), gambling for money and enhancement are usually the most common motives whereas the escape motive is one of the least endorsed motives, but there are some differences between countries.

Gambling motives have been shown to vary between socio-demographic and gambling behavior related factors (Francis et al., 2015; Hagfors et al., 2022; Pallesen et al., 2020; Volberg et al., 2015; Wardle et al., 2011). For example, gambling for money and enhancement is usually more common among men, whereas gambling for escape is more common among women (Francis et al., 2015; Pallesen et al., 2020; Wardle et al., 2011). Furthermore, gambling for socializing, enhancement and escape is more common among younger gamblers, while supporting worthy causes is more common among older gamblers (Francis et al., 2015; Pallesen et al., 2020; Volberg et al., 2015; Wardle et al., 2011). Some of the motives (e.g. escape, money, enhancement) have been associated with problem gambling more strongly than others (Abbott et al., 2018; Francis et al., 2015, 2015; Pallesen et al., 2020; Volberg et al., 2015).

Online gambling is currently changing gambling globally (Håkansson, 2020; King et al., 2020; Oksanen et al., 2021; Sirola et al., 2021). This emerging and transforming context of gambling involves also potentially different motives for gambling than traditional forms of offline gambling. Online gambling has been associated with escape motives and negative affect, and it is more often initiated to make money, to demonstrate skills or because of boredom than offline gambling (Goldstein et al., 2016). Although online gambling has been associated with problem gambling, the likely reason behind it could be that online gamblers often engage in multiple gambling forms, which is a strong predictor of problem gambling in itself (Abbott et al., 2018).

### Present Study

This study focused on the offshore and onshore online gamblers in Finland. Currently, a state-owned monopoly supplier Veikkaus Oy has exclusive right to provide gambling services in Mainland Finland. In 2017, three gambling operators (RAY, Fintoto and Veikkaus) were merged in order to prevent competition between the monopoly operators, and it was thought that monopoly supplier could be better at preventing and reducing gambling-related harms than licensed operators who compete with each other (Salonen et al., 2019). The Sect. 52 of the Lotteries Act regulates gambling in Mainland Finland. The Ministry of Interior governs gambling with a decree which regulates specific details in gambling provision, e.g., the wins paid to players and the maximum number of slot machines. The National Police Board monitors the provision of gambling services. The Lotteries Act has assigned the Ministry of Health and Social Affairs to monitor and research gambling harms which allocated this mission to the Finnish Institute of Health and Welfare. Veikkaus Oy must compensate the state for the costs incurred by monitoring, research, and development. Gambling profits are used to support public interest activities from health, culture, research, and sports fields. (The Sect. 52 of the Lotteries Act.)

To gamble onshore games provided by Veikkaus Oy, the customer is obligated to create a player account and verify their identity and age. Each customer can have only one account and must set limits to daily and monthly money transfer in order to gamble. Despite the monopoly, participating in offshore gambling, that is, games offered by Paf (Ålands Penningautomatförening) and/or foreign gambling operators, is not prohibited under Finnish legislation. Monopoly operator Paf operates in the Åland Islands by offering online games and games on ships sailing between Finland and Sweden or Estonia. Overall, the proportion of offshore gamblers has increased in Finland from 5.1 to 6.2% between 2015 and 2019 (Salonen et al., 2020). The most popular offshore game types in Finland were EGMs, online betting, and poker.

This study examines both gambling motives in general and the specific reasons for offshore gambling in Finland. Our research questions were:

1. How do gambling motives differ between offshore and onshore online gamblers?
2. What are the specific reasons for offshore gambling?

## Method

### Participants

The data from the Finnish Gambling 2019 population study was used for the present study (Salonen et al., 2020). The survey was assigned to the Finnish Institute of Health and Welfare and funded by the Finnish Ministry of Social Affairs and Health according to the Sect. 52 of the Lotteries Act. The purpose of the study is to monitor and explore Finn’s gambling, problem gambling, and attitudes and opinions towards gambling. The target population of the survey was 15–74 aged Finnish people living in Mainland (*N* = 7,800). Statistics Finland collected the data using computer-assisted telephone interviews. 3,994 Finns participated in the survey in 2019 (50.7% women, response rate 52%). Mean age of the participants was 45.0 years (*SD* = 17.1 years). Only those participants, who had gambled online during previous 12 months, were selected to this study. Underage participants (15–17 years old) where excluded from the analysis as they gamble mostly land-based games and particularly scratch cards and electronic gaming machines (Salonen et al., 2020). Five participants were excluded from the analysis due to missing information leaving a total of 1,422 participants to the final sample.

### Measures

Our study was focused on offshore gambling. The survey included questions on participating in 18 different game types provided by Veikkaus Oy, Paf or other offshore gambling operators and based on these we created a dichotomous variable indicating onshore and offshore gamblers (0 = *onshore gambler* and 1 = *offshore gambler*). Those participants who had gambled only games provided by Veikkaus Oy were categorized as onshore gamblers, and those participants who had gambled online games provided by offshore gambling operators, in addition to games provided by Veikkaus Oy, were categorized as offshore gamblers. Only a very few participants (*n* < 5) gambled on offshore gambling sites only, so they could not be analyzed separately. Online games provided by Paf were also categorized as offshore gambling since Paf is not a part of gambling monopoly system in Mainland Finland.

Gambling motives were asked with a categorical question: “What would you say is the main reason that you gamble?” (Williams et al., 2017). The answer options included: (1) for excitement, entertainment or fun, (2) to win money, (3) to improve your skills, (4) to compete with others or to challenge yourself, (5) to socialize, (6) to support worthy causes, (7) to escape, relax or to relieve stress, (8) because it makes you feel good about yourself, (9) other reason, and (10) do not know. At first, the participants were asked to choose only one primary motive for their gambling (main motive). Then, the participants were instructed to choose what secondary reasons they had for their gambling (additional motives).

The responses for both questions were recoded into seven categories based on Massachusetts Baseline Population Survey (SEIGMA, Volberg et al., 2015; see also Hagfors et al., 2022). The categories were: 1) Positive feeling, 2) Money, 3) Socializing, 4) Supporting worthy causes, 5) Escape, 6) Challenge, and 7) Other. The category ‘Positive feeling’ were combined from the options “For excitement, entertainment or fun” and “Because it made you feel good about yourself”. The options “to improve your skills” and “to compete with others or to challenge yourself’ were combined to form category ‘Challenge’, and the options “other” and “do not know” where combined, as well. Those participants who had indicated gambling on offshore gambling sites were also asked with an open-ended question what their reasons were to gamble on offshore sites (“What were your main reasons for gambling on sites other than Veikkaus Oy?”). All offshore gamblers answered to this question.

For the logistic regression models, the main motive and additional motives were combined to reflect gambling motives more broadly. Therefore, a simple term ‘motive’ is used when main motive and additional motive for gambling are considered together further in the text. Each motive was used as a dummy variable in the model. Challenge motive was not included in the models due to too small frequency. The amount of gambling motives was calculated from the previously mentioned motive categories and ranged between 0 and 6. We recoded the variable into three categories: ‘0–1 motive’, ‘2 motives’ and ‘ ≥ 3 motives’.

Problem gambling severity was measured with the Problem Gambling Severity Index (PGSI, Ferris & Wynne, 2001). PGSI is a self-report instrument including nine items which has been widely used for assessing problem gambling population surveys (Currie et al., 2013; Holtgraves, 2008). The PGSI items have four-point scale where 0 = *never*, 1 = *sometimes*, 2 = *most of the time*, 3 =* almost always*. The total scores range between 0 and 27 scores and it is treated as a continuous variable in the analysis. McDonald’s omega for the scale was excellent (ω = 0.89). One missing value was recoded as 0 after checking the weekly gambling expenditure (2.50 €), gambling frequency (1‒3 times in a month) and the number of game types gambled (1).

Gambling frequency was inquired for 18 pre-defined game types. The response options were ‘daily or several times per week’, ‘once a week’, ‘1‒3 three times per month’ and ‘less than monthly’. The overall gambling frequency was calculated based on the game type the respondent gambled most often. Furthermore, respondents were inquired how much money they used for gambling weekly, monthly or yearly. Weekly gambling expenditure was calculated based on these answers. Finally, age and gender were inquired from all respondents. The participants were divided into four age groups: 18‒29, 30‒39, 40‒54 and 55 years or older. As offshore gambling was much rarer among the oldest participants, we decided to put all the participants 55 or over into the same age group.

### Methods

We used binary logistic regression to analyze the associations between offshore gambling (dependent variable), gambling motives and gambling behavior related variables. The effects of the independent variables are presented as regression coefficients (B) and their standard errors (SE), odds ratios (OR) and their 95% confidence intervals. We also report Wald *χ*^*2*^ -coefficients and *p*-values for statistical significance based on the test. Data-driven qualitative content analysis was used to analyze the reasons why participants chose to gamble on offshore gambling websites. Coding structure was conducted by two authors independently without knowing each other. After the coding structure was set, both authors read the data independently. Third author then conducted the statistical inter-rater reliability analysis. Cohen’s kappa values varied from *κ* = 0.86, *p* < .001 [Benefits, bonuses, and the usability of the website] to *κ* = 1.00, *p* < .001 [Responsibility and Social reasons] indicating high inter-rater reliability.

## Results

### Statistical Results

Almost 85% of the sample were onshore gamblers while 15% gambled also on offshore gambling sites. Larger proportion of men than women gambled online, but the distinction was even sharper among offshore gamblers than among onshore gamblers (Table 1). Offshore gamblers also tended to be younger than those who gambled onshore only. What comes to gambling motives, gambling for positive feeling, socializing and escape were more common among offshore gamblers, whereas gambling for money or supporting worthy causes were more common among onshore gamblers. In addition, offshore gamblers tended to have more different motives and gamble more frequently than onshore gamblers. Finally, the average PGSI score was higher for offshore gamblers than onshore gamblers (1.88 vs. 0.24, *t* = -8.00, *p* < .001) and the same was true with the average weekly gambling expenditure (58.4€ vs. 8.41€, *t* = -3.25, *p* < .001).

**Table 1:** Descriptive statistics and the comparison of onshore and offshore online gamblers (n = 1,422)

	Onshore gamblers^a^ (*n* = 1,208)	Offshore gamblers^b^ (*n* = 214)	Pearson χ^*2*^	All (*n* = 1,422)
	*% (n)*	*% (n)*	*p*	*% (n)*
*Gender*			< 0.001	
Men	59.1 (723)	83.3 (179)		63.1 (902)
Women	40.9 (485)	16.7 (35)		36.9 (520)
*Age* (range 18–74)			< 0.001	
18–29 years	14.7 (146)	47.9 (94)		20.2 (240)
30–39 years	19.7 (217)	27.5 (60)		21.0 (277)
40–54 years	33.6 (403)	18.3 (43)		31.0 (446)
≥ 55 years	32.1 (442)	6.3 (17)		27.8 (459)
*Gambling motives*				
Positive feeling	53.8 (640)	73.2 (156)	< 0.001	57.0 (796)
Money	78.7 (955)	69.2 (149)	< 0.001	77.2 (1104)
Socializing	7.9 (89)	13.8 (28)	0.003	8.8 (117)
Worthy causes	12.2 (151)	4.6 (10)	< 0.001	10.9 (161)
Escape	3.3 (40)	11.3 (23)	< 0.001	4.6 (63)
*Amount of motives*			< 0.001	
0–1 motive(s)	37.1 (455)	23.4 (51)		34.9 (506)
2 motives	56.2 (673)	58.6 (125)		56.6 (798)
≥ 3 motives	6.7 (80)	18.0 (38)		8.6 (118)
*Gambling Frequency*			< 0.001	
Less than monthly	27.6 (319)	14.2 (29)		25.4 (348)
1–3 times/month	28.3 (332)	33.5 (70)		29.1 (402)
At least once/week	44.1 (557)	52.3 (115)		45.4 (672)

Note. ^a^Gambled only game types provided by Veikkaus Oy. ^b^Gambled both game types provided by Veikkaus Oy and/or offshore gambling operators

According to the logistic regression model (Table 2), men had higher odds to gamble on offshore sites than women confirming the descriptive results in the Table 1. Nagelkerke pseudo R^2^ for the model was 0.39. Younger age groups had multiple times higher odds to gamble on offshore sites than the participants over 55 years. What comes to gambling motives, offshore gamblers were less likely to gamble for money or to support worthy causes but had multiple reasons to gamble, when compared to onshore gamblers. Furthermore, offshore gambling was more common for those who gambled regularly than for those who gambled less often than once a month. Lastly, having gambling problems was more likely for offshore gamblers than for those who gambled onshore only. Although the average weekly gambling expenditure was significantly higher for offshore than onshore gamblers in the descriptive results, it was non-significant in the model.

**Table 2:** Predictors of offshore gambling among past-year online gamblers (n = 1,422)

	*B*	*SE (B)*	*Wald*	*OR*	95% *CI*
*Gender* (Ref. women)	0.91	0.21	18.64	2.49^***^	1.65–3.76
*Age* (Ref. ≥ 55 years)					
18–29 years	2.85	0.33	72.02	17.33^***^	8.97–33.48
30–39 years	1.95	0.34	33.87	7.02^***^	3.64–13.53
40–54 years	1.09	0.36	10.60	2.99^***^	1.55–5.77
*Positive Feeling*	-0.09	0.25	0.13	0.91	0.56–1.50
*Money*	-0.61	0.24	6.37	0.54^*^	0.34–0.87
*Socializing*	-0.62	0.34	3.35	0.54	0.28–1.03
*Worthy Causes*	-1.44	0.43	11.47	0.24^**^	0.10–0.55
*Escape*	-0.43	0.40	1.11	0.65	0.29–1.45
*Number of motives* (Ref. 0‒1 motive)					
2 motives	0.48	0.28	3.03	1.62	0.94–2.79
≥ 3 motives	1.85	0.50	13.74	6.35^***^	2.39–16.87
*Gambling frequency* (Ref. less than once/month)					
1–3 times/month	0.91	0.25	13.67	2.48^***^	1.53–4.01
At least once/week	1.08	0.25	17.94	2.93^***^	1.78–4.82
*Gambling expenditure*	0.01	0.00	2.32	1.01	1.00–1.01
*Problem Gambling* ^*a*^	0.38	0.07	32.88	1.46^***^	1.28–1.66

Note. *OR* = odds ratio; *CI* = confidence interval. ^a^Problem Gambling Severity Index, PGSI

^*^p < .05, ^**^p < .01, ^***^p < .001

### Qualitative Results

Offshore gamblers (n = 214) were asked with an open-ended question what their reasons were to gamble on offshore gambling sites. Six different categories were formed based on these answers (Table 3). The largest category was named ‘Larger game supply and game features’. This category included responses such as interesting and larger game supply, bigger payouts, and faster-paced games. The second largest category was named ‘Benefits, bonuses, and the usability of the website’ and included responses such as “easy to start gambling”, customer benefits and “the winnings were paid fast”. The third largest category was named ‘Inner motivation’ and included responses such as curiosity and time passing. ‘Social reasons’ included responses such as a friend recommended or pastime with friends. The category ‘Responsibility’ refers to the responsible gambling procedures the gambling sites use, such as offering consumer protection tools. Some of the respondents indicated that they gambled on offshore gambling sites because the onshore sites used these consumer protection tools. A few respondents indicated that they did not want to support the monopoly status of Veikkaus Oy and therefore used the offshore gambling websites. Finally, the last category was ‘Marketing’ which included advertising, for example. Four answers were excluded as their meaning was not clear or they did not fall into any category (the respondent had invested to the gambling operator, for example).

**Table 3:** Reasons to gamble on offshore gambling websites (n = 214)

Category	*n*	*%*
Larger game supply and game features	164	76.3
Benefits, bonuses, and the usability of the website	72	33.5
Inner motivation	40	18.6
Social reasons	21	9.8
Responsibility	16	7.4
Marketing	10	4.7

## Discussion

This study investigated the general gambling motives of onshore and offshore online gamblers, and the specific reasons to gamble on offshore gambling sites. According to our results, offshore gamblers gambled rarely for money or worthy causes but had more different motives to gamble than onshore gamblers. Offshore gamblers tended to gamble more frequently and had higher weekly gambling expenditure than onshore gamblers, although the difference in the gambling expenditure was no longer significant in the logistic regression model.

### Gender and age Differences

As expected, offshore gambling was more common among men. This finding is in line with Gainsbury and her colleagues (2018): offshore gambling is more common among men than women. This finding might be explained by the fact that men gamble more often than women overall. Furthermore, the most game types that women often prefer (e.g., EGMs and lotteries) are already offered on the onshore gambling sites (Salonen et al., 2020). Women also gamble online less than men in general (McCormack et al., 2014; Salonen et al., 2020). Moreover, offshore gamblers had significantly higher PGSI scores than onshore gamblers. Problem gambling is known to be the most prevalent among young men (Salonen et al., 2020). According to the annual report of national helpline Peluuri, young adult males were the largest group to contact helpline and chat services, and the role of offshore gambling in gambling problems has continued to grow (Silvennoinen & Vuorento, 2021). The high gambling expenditure combined with low income associated with studying or early career stage may pose young men at heightened risk for gambling problems as the buffer against financial problems is weaker (Castrén et al., 2018).

The finding that offshore gambling was more common among younger gamblers confirms the previous findings (Costes et al., 2016; Gainbury et al. 2018; 2019), as well. Younger generation has grown up surrounded by internet in their daily life and easily adopt new technologies (Ahn, 2011). They probably feel more confident exploring new gambling forms on offshore gambling websites than older generation who might feel less confident in online environment, and worry about becoming a victim of fraud (Burton et al., 2022). Furthermore, youth is a period when seeking novel experiences and risk taking is common (Oksanen et al., 2017, 2018; Sussman & Arnett, 2014). Those youths who already hold positive attitudes towards gambling may find new gambling content encountered on social media especially appealing (Kaakinen et al., 2020). Gambling marketing has increased, and people can be exposed to gambling advertising even without searching any related information (Guillou-Landreat et al., 2021). Large amount of the gambling marketing is connected to sports and is designed to strengthen the mental association of sports and gambling, sometimes referred as sportification of gambling. This type of advertising is targeted especially to young men, and depicts gambling as exciting, fun, and effortless lifestyle.

### Motive Types and Multiple Motives

The result that gambling for money was less common for offshore gamblers than onshore gamblers was surprising given that online gambling is often initiated to make money (Goldstein et al., 2016). Offshore gamblers might be more motivated by the mood-altering effects of games or social reasons, as was shown in the descriptive results although the difference was no longer statistically significant in the model. Offshore gambling sites may provide opportunities to gamble with higher risk which in turn increases the thrill and excitement (Baudinet & Blaszczynski, 2013; Rockloff et al., 2007). Young people with sensation seeking and impulsive behavior may find high risk games especially appealing which places them at higher risk for developing gambling problems as a result. However, the result that supporting worthy causes was less common for offshore gamblers was expected since the offshore gambling operators rarely use their profits to common-good purposes.

The results also revealed that offshore gamblers had more different motives, gambled more often, spent more money on gambling, and had higher PGSI scores than onshore gamblers. It is likely that all these factors represent the intensity of gambling which is inherently related to problem gambling (Abbott et al., 2018; Binde et al., 2017; Ferris & Wynne, 2001). This is in accordance with previous studies which found out that offshore gambling is associated with more intense gambling patterns and problem gambling (Costes et al., 2016; Gainsbury et al., 2018; Hing et al., 2021). If gambling has a big role in one’s life, it’s meaning may also be manyfold so that it can fulfill several different needs. It is likely that those who already have gambling problems seek new and more thrilling gambling experiences from offshore gambling sites, but also using offshore gambling sites can facilitate more intensive gambling and accelerate the development of problem gambling. The motives for gambling may also differ in the different phases of the addiction: pleasure is important at the onset of the addiction but eventually mood regulation, loss of control and changes in tolerance become central (APA, 2013; Blaszczynski et al., 2008).

### Reasons for Choosing Offshore Gambling Sites

The results from the qualitative analysis revealed that offshore gamblers have multiple different reasons for choosing offshore gambling sites. The most important reasons were larger game supply and game features which corresponds the previous findings (Gainsbury et al., 2018, 2019). This finding suggests that some gamblers are not satisfied with the onshore game supply and gambling experiences, which may push them to search new gambling experiences elsewhere. Offshore gambling sites can provide larger variation of game features, as they do not follow the same restrictions and consumer protection requirements as onshore sites. The second most common reasons to gamble on offshore gambling sites were benefits, bonuses, and the usability of the website, which are designed to appeal new customers and to commit the existing ones. This finding is also in accordance with the study by Gainsbury et al. (2019).

### Implications for Policy and Practice

The Lotteries Act that regulates gambling in Finland will be reformed (Ministry of Social Affairs and Health, 2022). The reform focuses on preventing harm caused by gambling, combating illegal marketing, and directing the demand for gambling towards activities that are covered by the Lotteries Act. Lotteries Act will include, for example, a new instrument which enables to block payment transactions to offshore gambling operators that direct their marketing to mainland Finland (Rydman & Tukia, 2019). This study provides important new knowledge that supports restricting offshore gambling. Offshore gambling poses a threat to public health and especially for young people with impulsive and risky behavior. This group should be targeted in preventive programs.

### Limitations

This study has some limitations that should be considered. First, the study is limited to Mainland Finland only excluding the immigrants, institutionalized person or those speaking other language than Finnish or Swedish. Second, the monopoly system in Finnish gambling regulation is somewhat uncommon compared to many other countries, which may weaken the generalizability of the study results into countries with different licensing systems. Third, the study design was cross-sectional denoting that no causal conclusions can be made. Future studies would benefit from using longitudinal study design. Finally, the study utilized self-reported data which enables possible social desirability bias where participants tend to answer in socially acceptable ways which does not wholly reflect their genuine reality (Krumpal, 2013). The participants may, for example, underreport some sensitive information, such as gambling severity or gambling expenditure.

## Conclusions

Offshore online gambling was more common among men and younger age groups than among women or older age groups. Offshore gamblers gambled rarely for money or supporting worthy causes, but they had multiple gambling motives, more intense gambling behavior, and more severe problem gambling than onshore gamblers. Offshore gambling poses a heightened risk for problem gambling especially for young men with impulsive and risky behavior. The most common reasons for choosing offshore gambling sites were larger game supply and game features implicating that some gamblers are not satisfied with the onshore game supply.

## Data Availability

The survey data without any register-based information is publicly accessible for research purposes from the Finnish Society Science Data Archive (FSD) with the name of Gambling Survey 2019 (ID: FSD3522; Persistent identifier: urn:nbn:fi:fsd:T-FSD3522).
